# The Role of Multimodality Cardiac Imaging in the Diagnosis and Management of Chronic Chagas Cardiomyopathy

**DOI:** 10.1016/j.jaccas.2025.105747

**Published:** 2025-11-19

**Authors:** Ana Carolina do A.H. de Souza, Miar Elaskandrany, Sara Rendell, Diana Lopez, David T. Martin, Jorge A. Alvarez

**Affiliations:** a Department of Medicine, Brigham and Women's Hospital, Boston, Massachusetts, USA; b Division of Infectious Diseases, Brigham and Women's Hospital, Boston, Massachusetts, USA; c Division of Cardiovascular Medicine, Brigham and Women's Hospital, Boston, Massachusetts, USA; d Cardiac Arrhythmia Service, Division of Cardiovascular Medicine, Brigham and Women's Hospital, Boston, Massachusetts, USA

**Keywords:** Chagas disease, chronic Chagas cardiomyopathy, left ventricular dysfunction, multimodality imaging

## Abstract

**Background:**

Chronic Chagas cardiomyopathy (CCC) is a late manifestation of *Trypanosoma cruzi* infection, now increasingly prevalent in nonendemic regions owing to migration. Diagnosis can be challenging, especially in patients with comorbid presentations.

**Case Summary:**

A 50-year-old man originally from El Salvador presented with subacute dyspnea, fever, and weight loss. Work-up revealed severe biventricular dysfunction, apical aneurysm, and pulmonary consolidation. Multimodality cardiac imaging characterized structural abnormalities and excluded alternative causes of dilated cardiomyopathy. Serologic testing confirmed the diagnosis of Chagas cardiomyopathy, and blood cultures grew *Francisella tularensis*. The patient received targeted antibiotics for tularemia and guideline-directed medical therapy for heart failure.

**Discussion:**

CCC causes progressive myocardial fibrosis, conduction abnormalities, and malignant arrhythmias. Advanced cardiac imaging—especially magnetic resonance imaging—facilitates detection of apical aneurysms and fibrosis. Implantable cardioverter-defibrillator placement is indicated for secondary prevention in CCC and is considered for primary prevention in high-risk patients. Coexistent infections may obscure presentation.

**Take-Home Messages:**

Suspect CCC in patients with new left ventricular dysfunction, especially in those with a travel history to endemic areas. Multimodality imaging helps characterize myocardial fibrosis/scar and exclude alternative etiologies. CCC carries high arrhythmic risk, and patients should be assessed for advanced therapies.

## Visual Summary

**Figure:**
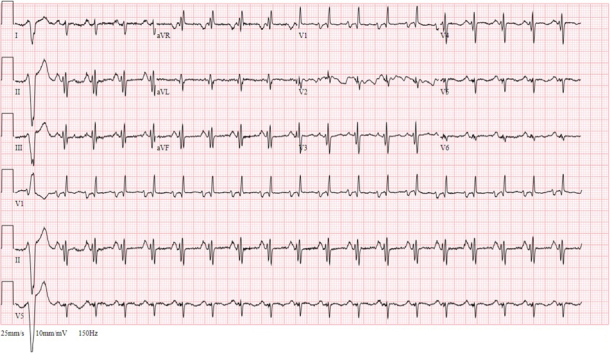

**Visual Summary:**
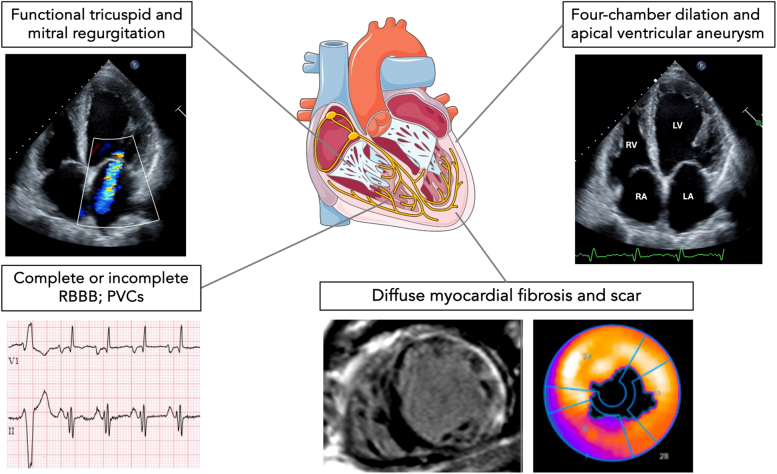
Clinical and Imaging Findings in Patients With Chronic Chagas Cardiomyopathy PVC = premature ventricular complex; RBBB = right bundle branch block.

## History of Presentation

A 50-year-old man from El Salvador currently living on the island of Nantucket, Massachusetts, presented to the local emergency department with fatigue and progressive dyspnea on exertion for 2 months. The symptoms had worsened 2 weeks before presentation, accompanied by cough, fevers, night sweats, and unintentional 8-pound (3.63-kg) weight loss. Initial vital signs in the emergency department showed tachycardia, fever (103.9 °F [39.94 °C]), and tachypnea. The physical examination was remarkable for diaphoresis, increased work of breathing, and diffuse coarse crackles on lung auscultation. Heart rhythm was regular, and no murmurs were appreciated. No lower extremity edema or abdominal tenderness was observed. The rest of the examination was unremarkable.

**Take-Home Messages**

• CCC should be considered among the differential diagnoses in patients presenting with new LV dysfunction, particularly those with a history of immigration or travel to endemic areas.
• Multimodality cardiac imaging enables the characterization of myocardial fibrosis and scar and can rule out other causes of myocardial inflammation.
• Patients with CCC are at higher risk for sudden cardiac death due to malignant ventricular arrhythmias and should be considered for advanced therapies.

**Figure 1:**
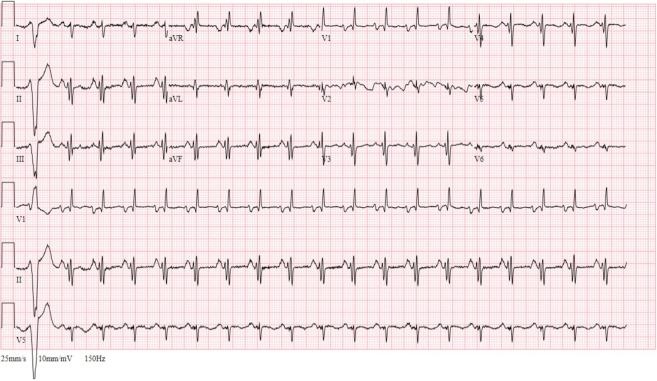
Electrocardiogram on Admission Electrocardiogram showed sinus tachycardia with premature ventricular complexes, biatrial enlargement, and incomplete right bundle branch block.

**Figure 2:**
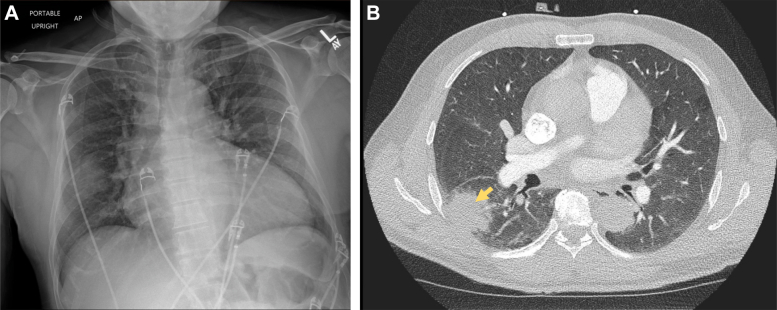
Chest Imaging Performed on Admission (A) Chest radiography showed enlarged cardiac silhouette, low lung volumes, and right mid-hemithorax opacity. (B) Chest computed tomography showed a right lower lobe subpleural consolidation with surrounding ground-glass opacities (yellow arrow), prominent mediastinal and bilateral hilar lymph nodes, and cardiomegaly with left ventricular enlargement.

A 12-lead electrocardiogram showed sinus tachycardia with premature ventricular complexes, biatrial enlargement, right ventricular hypertrophy, and incomplete right bundle branch block (Figure 1). Chest radiography revealed an enlarged cardiac silhouette, diffuse pulmonary edema, and focal airspace opacity in the right midlung (Figure 2A). A bedside ultrasound showed severely depressed left ventricular (LV) function and plethoric inferior vena cava, and a decision was made to transfer the patient to a tertiary hospital.

## Past Medical History

The patient had no significant medical history. He worked in landscaping and had not seen a doctor in the past decade. He was born in El Salvador, had immigrated to the United States 25 years before presentation, and had traveled to El Salvador for 1 month in the past year. He denied any known insect or tick bites but endorsed frequent contact with rabbit carcasses at work. There was no history of incarceration, injected drug use, blood transfusion, immunosuppression, or family history of sudden cardiac death.

## Differential Diagnosis

The differential diagnoses for a patient presenting with new severe LV dysfunction and dyspnea is broad and includes cardiovascular causes such as ischemic heart disease, undiagnosed valvular disease, idiopathic dilated cardiomyopathy, and alcohol cardiomyopathy. Given that the patient had fever, unintentional weight loss, and a pulmonary consolidation, other considerations included infectious (HIV-associated cardiomyopathy, viral myocarditis, and/or pericarditis) and malignant etiologies.

## Investigations

**Figure 3:**
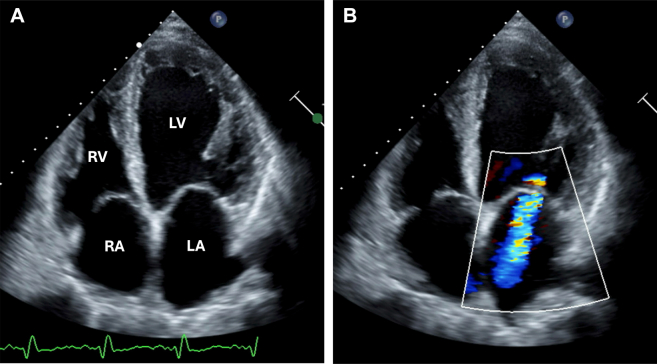
Transthoracic Echocardiography Findings (A) Transthoracic echocardiography showed severe dilation of the left ventricle (LV), with severely reduced LV systolic function (ejection fraction: 20%). The right ventricle (RV) was mildly dilated with mildly reduced function. The left atrium (LA) and right atrium (RA) were dilated. (B) There was moderate mitral and tricuspid regurgitation with color Doppler shown over the mitral valve.

Initial laboratory evaluation was notable for elevated N-terminal pro–B-type natriuretic peptide, elevated and adynamic high-sensitivity troponin-T, and mild leukocytosis. A computed tomography (CT) pulmonary angiogram showed no pulmonary embolism but revealed a right lower lobe subpleural consolidation with surrounding ground-glass opacities (Figure 2B). Esophageal thickening was incidentally found. Transthoracic echocardiography (TTE) revealed severe LV dilation and systolic dysfunction, with an ejection fraction of 20%. There was mildly reduced right ventricular systolic function and moderate tricuspid and mitral valve regurgitation (Figure 3). Given extended time spent in El Salvador and pulmonary consolidation, a comprehensive infectious work-up was ordered. This included blood cultures, HIV 1,2 fifth-generation screen, Lyme disease, *Babesia*, *Anaplasma*, viral hepatitis, and dengue, Zika, and chikungunya fever serologies, all of which were initially negative. Active tuberculosis was ruled out via 3 negative acid-fast bacilli stain with negative *Mycobacterium tuberculosis* complex polymerase chain reaction from serial sputum samples. Blood parasite smear was negative. A *Trypanosoma cruzi* antibody was sent and remained pending during the initial days of the hospitalization.

**Figure 4:**
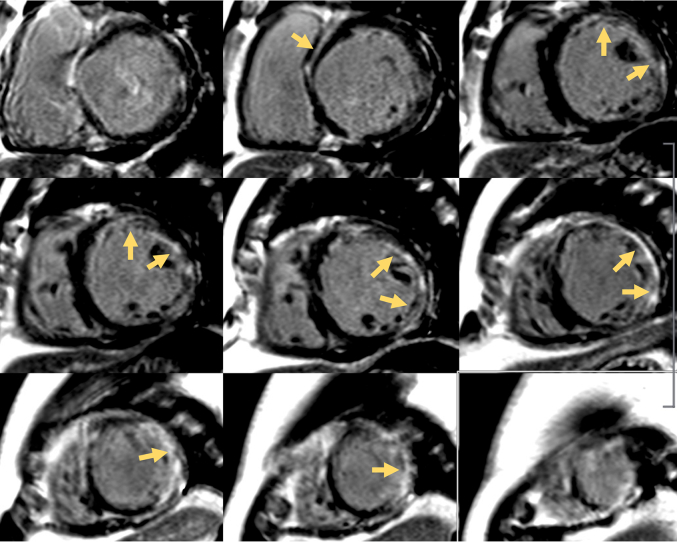
Sequential Cardiac Magnetic Resonance Imaging Findings Cardiac magnetic resonance imaging showed an extensive amount of late gadolinium enhancement involving the basal midwall septum, subendocardial basal anterior and anterolateral walls, midwall involving the mid anterolateral and inferolateral wall, and transmural apical lateral wall and apex (yellow arrows).

**Figure 5:**
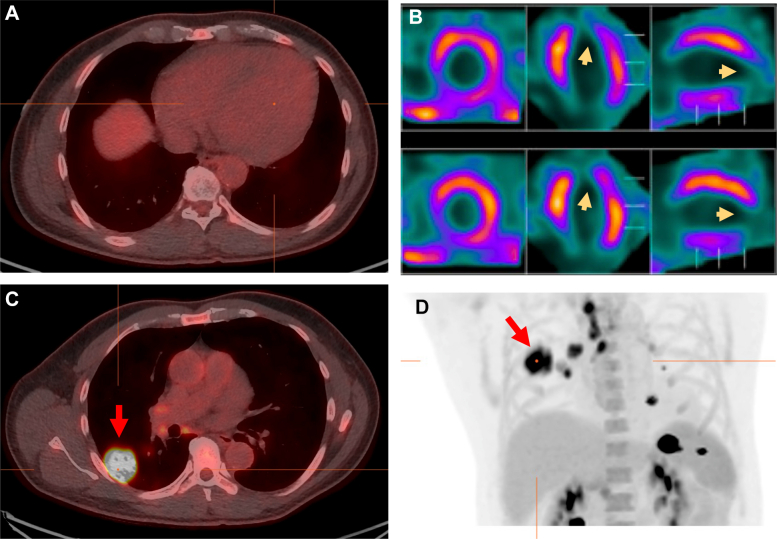
Cardiac FDG PET/CT Images (A and D) Cardiac FDG PET/CT after a high-fat low-carbohydrate diet showed no focal myocardial uptake. (B) There was focal myocardial scar involving the apex and the apical lateral walls (yellow arrows). (C and D) Intensely FDG-avid right lower lobe subpleural consolidation, likely representing an infectious etiology, was observed (red arrow). FDG = fluorodeoxyglucose; PET/CT = positron emission tomography/computed tomography.

To rule out obstructive epicardial coronary artery disease, a coronary CT angiography was performed, which revealed minimal stenosis (<25%) of the proximal left anterior descending artery, diagonal branches, and right coronary artery. Sequential cardiac magnetic resonance imaging (MRI) showed severe LV dilation with an apical aneurysm and extensive LV late gadolinium enhancement without evidence of myocardial edema on T2-weighted imaging but with diffuse native T1 times and extracellular volume fraction in the areas of late gadolinium enhancement (Figure 4). Findings were concerning for a fibrotic myocardial pathology. Cardiac fluorodeoxyglucose (FDG) positron emission tomography (PET)/CT was performed and showed no evidence of focal myocardial inflammation after an adequate high-fat low-carbohydrate diet (Figure 5). There was a focal myocardial scar involving the apex and the apical lateral walls, corresponding to the areas of transmural fibrosis on MRI. An FDG-avid right lower lobe subpleural consolidation with mediastinal, hilar, and supraclavicular lymphadenopathy was observed, highly suspicious for infection (Figure 5B).

## Management

Given the initial concern for bacterial pneumonia, the patient was empirically started on doxycycline and ceftriaxone. He also received intravenous furosemide for pulmonary edema with respiratory distress. Once euvolemic, he was started on guideline-directed medical therapy. He continued empiric antibiotic therapy until hospitalization day 4, when blood cultures resulted with small, Gram-negative coccobacilli consistent with *Francisella tularensis* at 5 days of incubation. Infectious disease consultants recommended targeted treatment for pulmonary tularemia with a 10-day course of ciprofloxacin. On hospitalization day 8, serologic testing resulted positive for *T.* *cruzi* IgG using 2 different, commercially available and Food & Drug Administration–validated assays (purified antigen assay, Hemagen Chagas Kit, Hemagen Diagnostics Inc and recombinant antigen assay, Chagatest ELISA recombinante v.3.0, Wiener Laboratories). When queried, the patient was not sure about prior contact with the triatomine insect.

The day before discharge, the patient experienced an episode of nonsustained ventricular tachycardia, during which he was hemodynamically stable. Given severe LV dysfunction and the risk of malignant arrhythmias, an implantable cardioverter-defibrillator (ICD) was recommended. He was also deemed to be at high risk for cardioembolic events given the presence of an LV aneurysm, and anticoagulation was discussed; however, both interventions were ultimately declined by the patient.

## Outcome and Follow-Up

After 11 days of hospitalization, the respiratory symptoms resolved. The patient was discharged on a maintenance diuretic dose, metoprolol succinate, sacubitril-valsartan, and empagliflozin, all of which were well tolerated. He was referred to a new primary care provider as well as to the heart failure and electrophysiology teams to continue medical management and discuss ICD implantation in the outpatient setting.

## Discussion

Chronic Chagas cardiomyopathy (CCC) affects 30% of patients infected by the protozoan *T.* *cruzi*, affecting over 6 million people worldwide, primarily in Central and South America.^1^ New patterns of migration have increased the prevalence of Chagas disease in nonendemic areas, posing a new diagnostic challenge to health care providers, who should be able to recognize this condition. Recent U.S. series have described a *T.* *cruzi* seroprevalence up to 16.5% in Latin American patients hospitalized for cardiac causes regardless of ejection fraction.^2^^,^^3^

CCC develops after decades of infection owing to an imbalance between parasite-driven and host immune reaction in the myocardial tissue, characterized by inflammatory cell infiltration, interstitial edema, fibrosis, and microvascular damage. Clinical manifestations stem from conduction abnormalities, severe LV dysfunction, and thromboembolic events. Electrophysiological abnormalities include complete or incomplete right bundle branch block, pathological Q waves, frequent premature ventricular complexes, and ventricular tachyarrhythmias, the main cause of sudden cardiac death in people with CCC.

TTE identifies functional and structural abnormalities and should be performed in all patients with suspected CCC. Common initial findings include segmental wall motion abnormalities, especially in the posterior and apical walls. As the disease progresses, global systolic dysfunction, atrial enlargement, and tricuspid and mitral regurgitation can occur,^4^ as seen in our patient. Apical aneurysms are present in two-thirds of patients with moderate to severe myocardial involvement and predict intramural thrombus formation and stroke risk. In these patients, ultrasound enhancing agents and off-axis views are important to allow for a detailed evaluation of the LV apex.

Additional imaging modalities are critical to better characterize myocardial composition and exclude other dilated cardiomyopathy causes. Coronary CT angiography can exclude obstructive epicardial disease. Importantly, cardiac MRI provides quantitative and qualitative assessment of myocardial fibrosis and enables risk stratification of patients at high risk for malignant arrhythmic events.^4^ Despite our patient not having a visible aneurysm on the noncontrast TTE, it was later identified on cardiac MRI, highlighting the importance of high clinical suspicion in at-risk populations. Although apical aneurysms are independent risk factors for stroke in patients with CCC, initiating anticoagulation in the absence of intramural thrombi remains controversial, and the decision should be individualized.^5^ Ultimately, FDG PET/CT can be used to differentiate other fibrotic diseases such as sarcoidosis or giant cell myocarditis. While absence of acute inflammation on cardiac FDG PET/CT does not fully exclude cardiac sarcoidosis, in the present case it redemonstrated focal uptake at the lung consolidation compatible with pulmonary tularemia, a rare disease caused by *F.* *tularensis* and associated with inhalation of aerosolized carcasses of infected animals.

In patients with acute *T.* *cruzi* infection, treatment with antitrypanosomal agents, including benznidazole or nifurtimox, results in clinical cure in 60% to 85% of patients. However, treatment does not provide a mortality benefit or slower disease progression in patients with established CCC, as demonstrated in the BENEFIT trial.^6^ Given the risk of reactivation in selected populations, treatment is recommended for immunosuppressed patients with reactivation of *T.* *cruzi*, such as transplanted patients and women of childbearing age.^5^^,^^7^^,^^8^ Current guidelines recommend medical therapy similar to other forms of heart failure, but larger Chagas-focused studies are needed. In patients with documented sustained ventricular tachycardia, ventricular fibrillation, or aborted sudden cardiac death, ICD placement is indicated for secondary prevention, with ongoing studies evaluating its role for primary prevention.^9^ In patients with recurrent sustained ventricular tachycardia, ablation with endoepicardial mapping may be required.^10^

Unfortunately, state-of-the-art imaging such as cardiac MRI is frequently unavailable in underserved areas where Chagas disease is endemic. Furthermore, patients with Chagas disease, who encounter systemic marginalization within health care systems, continue to face inequitable access to screening and diagnostic tools in resource-abundant nonendemic areas. Systemic efforts are warranted to address the growing structural barriers experienced by affected communities.

## Conclusions

Chagas disease affects a growing population in the United States and remains underdiagnosed because of barriers to accessing care in at-risk populations and lack of familiarity from providers. CCC should be considered in individuals from endemic areas presenting with new dilated cardiomyopathy of unclear etiology. A multidisciplinary approach is vital to diagnose and adequately treat CCC and its comorbid conditions. When available, cardiac imaging helps to determine the disease patterns, mainly characterized by myocardial fibrosis, and rule other causes leading to myocardial inflammation and scar.

## Funding Support and Author Disclosures

The authors have reported that they have no relationships relevant to the contents of this paper to disclose.

## References

[1] (2015). Chagas disease in Latin America: an epidemiological update based on 2010 estimates. Wkly Epidemiol Rec.

[2] Kerai A., Gadodia R., Aberra T. (2024). Seroprevalence of Chagas cardiomyopathy among hospitalized Latin American immigrants within a Washington, DC, Hospital. JACC Heart Fail.

[3] Irish A., Whitman J.D., Clark E.H., Marcus R., Bern C. (2022). Updated estimates and mapping for prevalence of Chagas disease among adults, United States. Emerg Infect Dis.

[4] Nunes M.C.P., Badano L.P., Marin-Neto J.A. (2018). Multimodality imaging evaluation of Chagas disease: an expert consensus of Brazilian Cardiovascular Imaging Department (DIC) and the European Association of Cardiovascular Imaging (EACVI). Eur Heart J Cardiovasc Imaging.

[5] Nunes M.C.P., Beaton A., Acquatella H. (2018). Chagas cardiomyopathy: an update of current clinical knowledge and management: a scientific statement from the American Heart Association. Circulation.

[6] Morillo C.A., Marin-Neto J.A., Avezum A. (2015). Randomized trial of benznidazole for chronic Chagas’ cardiomyopathy. N Engl J Med.

[7] Pan American Health Organization (2019). Guidelines for the Diagnosis and Treatment of Chagas Disease. https://iris.paho.org/handle/10665.2/49653.

[8] Forsyth C.J., Manne-Goehler J., Bern C. (2022). Recommendations for screening and diagnosis of Chagas disease in the United States. J Infect Dis.

[9] Martinelli M., Rassi A., Marin-Neto J.A. (2013). CHronic use of Amiodarone aGAinSt Implantable cardioverter-defibrillator therapy for primary prevention of death in patients with Chagas cardiomyopathy Study: rationale and design of a randomized clinical trial. Am Heart J.

[10] Romero J., Velasco A., Pisani C.F. (2021). Advanced therapies for ventricular arrhythmias in patients with Chagasic cardiomyopathy: JACC state-of-the-art review. J Am Coll Cardiol.

